# Preparation and characterization of nano liposomes of *Orthosiphon stamineus* ethanolic extract in soybean phospholipids

**DOI:** 10.1186/1472-6750-14-23

**Published:** 2014-03-27

**Authors:** Abdalrahim FA Aisha, Amin Malik Shah Abdul Majid, Zhari Ismail

**Affiliations:** 1 Department of Pharmaceutical Chemistry, School of Pharmaceutical Sciences, Universiti Sains Malaysia (USM), Minden 11800, Pulau Pinang, Malaysia; 2 Department of Pharmacology, School of Pharmaceutical Sciences, Universiti Sains Malaysia (USM), Minden 11800, Pulau Pinang, Malaysia; 3 Department of Pharmacy, School of Medicine and Health Sciences, An Najah National University, Nablus, West Bank, Palestine

**Keywords:** *Orthosiphon stamineus*, Soybean lecithin, Soybean phospholipids, Liposomal drug delivery system

## Abstract

**Background:**

*O. stamineus* is a medicinal herb with remarkable pharmacological properties. However, poor solubility of the active principles limits its medicinal value. This study sought to prepare nano liposomes of OS ethanolic extract in unpurified soybean phospholipids in order to improve its solubility and permeability. OS liposomes were prepared by the conventional film method, and were characterized for solubility, entrapment efficiency, Fourier transform infrared spectroscopy (FTIR), transmission electron microscopy (TEM), particle size and zeta potential, release, absorption in everted rat intestinal sacs, and DPPH scavenging effect.

**Results:**

OS liposomes showed substantial enhancement of extract’s solubility from 956 ± 34 to 3979 ± 139 μg/ml, with entrapment efficiency of 66.2 ± 0.9%. FTIR study indicates interaction between soybean phospholipids and OS extract. TEM and dynamic light scattering showed presence of round anionic nano liposomes with particle size and zeta potential of 152.5 ± 1.1 nm and −49.8 ± 1.0 mV, respectively. A study using the fluorescent probe pyrene showed the critical micellar concentration is 9.2 ± 2.9 μg/ml. Release studies showed 94 ± 0.1% release in non-formulated extract and 62.4 ± 0.1% in OS liposomes. Released extract from OS liposomes showed improvement in DPPH scavenging effect, IC50 = 23.5 ± 1.1 μg/ml compared to 32.4 ± 0.5 μg/ml in non-formulated extract. OS liposomes were stable at pH 5.5 and 7.4, but showed reversible agglomeration at pH 1.6. Absorption in everted rat intestinal sacs showed substantial improvement in permeability of 3′-hydroxy-5, 6, 7, 4″-tetramethoxyflavone, sinensetin, eupatorin, and 3 other unknown compounds.

**Conclusions:**

Enhanced solubility, absorption and antioxidant effect may improve the overall pharmacological effects and medicinal value of OS ethanolic extract.

## Background

**Figure 1:**
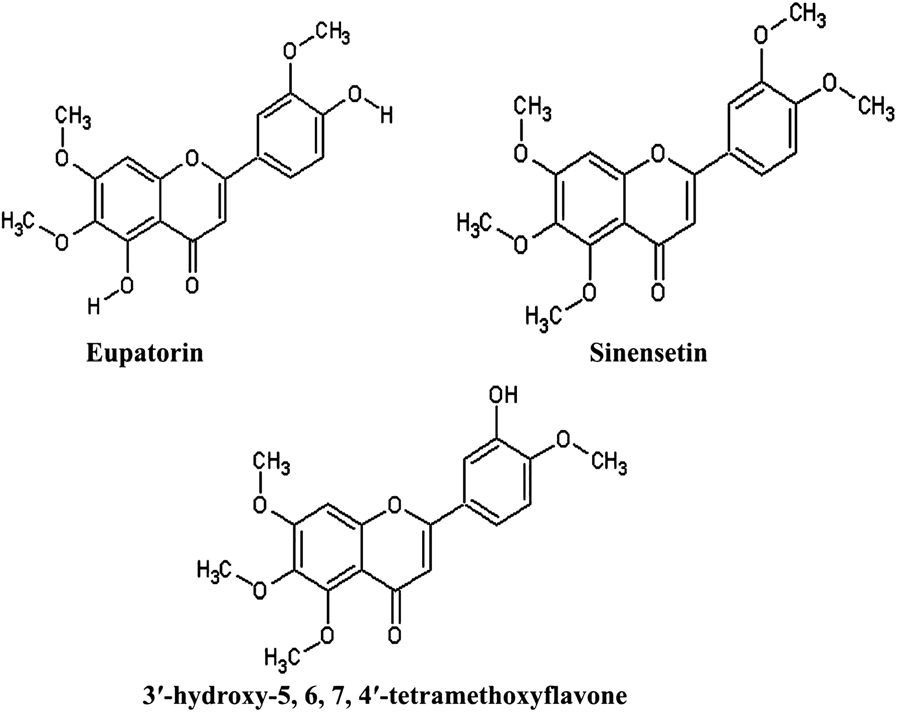
Chemical structure of 3′-hydroxy-5, 6, 7, 4′-tetramethoxyflavone (TMF), sinensetin (SIN) and eupatorin (EUP).

*Orthosiphon stamineus* Benth. (Lamiaceae) is a medicinal herb widely distributed in Southeast Asia. Leaves of this plant are commonly used in Southeast Asia and Europe as herbal tea. OS is used as a traditional medicine for treatment of some angiogenesis- related diseases such as rheumatism, tumorous edema, obesity, diabetic retinopathy and psoriasis [1]. Research on OS has increased recently due to its various pharmacological properties such as diuretic and hypouricemic [2], hepatoprotective [3], anti-hyperglycemic [4], antioxidant and antimicrobial [5-7], antiapoptotic [8], antiangiogenic and anticancer effects [1]. Pharmacological effects of OS are attributed to presence of polyphenolics, glycosides, lipophilic flavones [9], rosmarinic acid (RA) and caffeic acid derivatives, triterpenes [10], and diterpens [11-13]. The lipophilic flavones of OS including sinensetin (SIN), eupatorin (EUP) and 3′-hydroxy-5, 6, 7, 4′-tetramethoxyflavone (TMF) (Figure 1) have been given considerable interest as markers of pharmacological activity by several researchers [1,9,14]. However; these lipophilic compounds suffer poor aqueous solubility which limits the OS oral bioavailability and therapeutic applications. The OS ethanolic extract is rich in the lipophilic flavones such as EUP, SIN and TMF while containing very low concentration of proteins and polysaccharides. Therefore, improving solubility of the lipophilic flavones may improve the bioavailability and hence the overall pharmacological activity of OS extracts.

Liposomes are artificial vesicles formed by one or more concentric lipid bilayers separated by water compartments [15]. They have a unique ability of encapsulating hydrophobic, hydrophilic and amphiphilic compounds [16,17]. Drugs with varying hydrophobicities can be encapsulated in the phospholipid bilayer, in the entrapped aqueous core, or at the bilayer interface [18]. Liposomal drug delivery systems have several advantages such as improving solubility, bioavailability and efficacy, reduced toxicity, and increased product stability and patient compliance [15,16,18]. Phospholipids from soybean lecithin are widely used in liposomal drug delivery systems due to their safety, and wide availability at relatively low cost for upscale production. Crude soybean lecithin contains 65–75% phospholipids, together with triglycerides and smaller amounts of other substances such as carbohydrates, pigments, sterols and sterol glycosides [19]. The major phospholipids in soybean lecithin are phosphatidylcholine (PC), phosphatidylethanolamine (PE), and phosphatidylinositol (PI) [20]. Liposomes of herbal extracts or isolated compounds are usually prepared in pure phospholipids [21,22], or in crude phospholipid extracts [17,23]. However, the cost of pure phospholipids is a limiting factor in upscale production of liposomal drug delivery systems particularly those containing herbal extracts or dietary supplements [24]. It was estimated that one kilogram of pure natural phospholipids costs around 980 Euro [17], whereas the cost of crude soybean phospholipids is only 5% of the pure one, which makes unpurified soybean phospholipids a good alternative and attractive choice.

This study sought to produce a cost effective nano liposomes of OS ethanolic extract in crude soybean phospholipids obtained from food grade soybean lecithin, in order to improve the extract’s solubility and permeability as major factors for improving oral bioavailability. The liposomes were prepared by the film method and were evaluated for solubility, entrapment efficiency, stability under various pH conditions, critical micellar concentration, particle size and zeta potential, FTIR spectroscopy, TEM, drug release, absorption through everted rat’s small intestinal sacs, and free radical scavenging effect.

## Results and discussion

### Analysis of soybean phospholipids and OS extract

**Figure 2:**
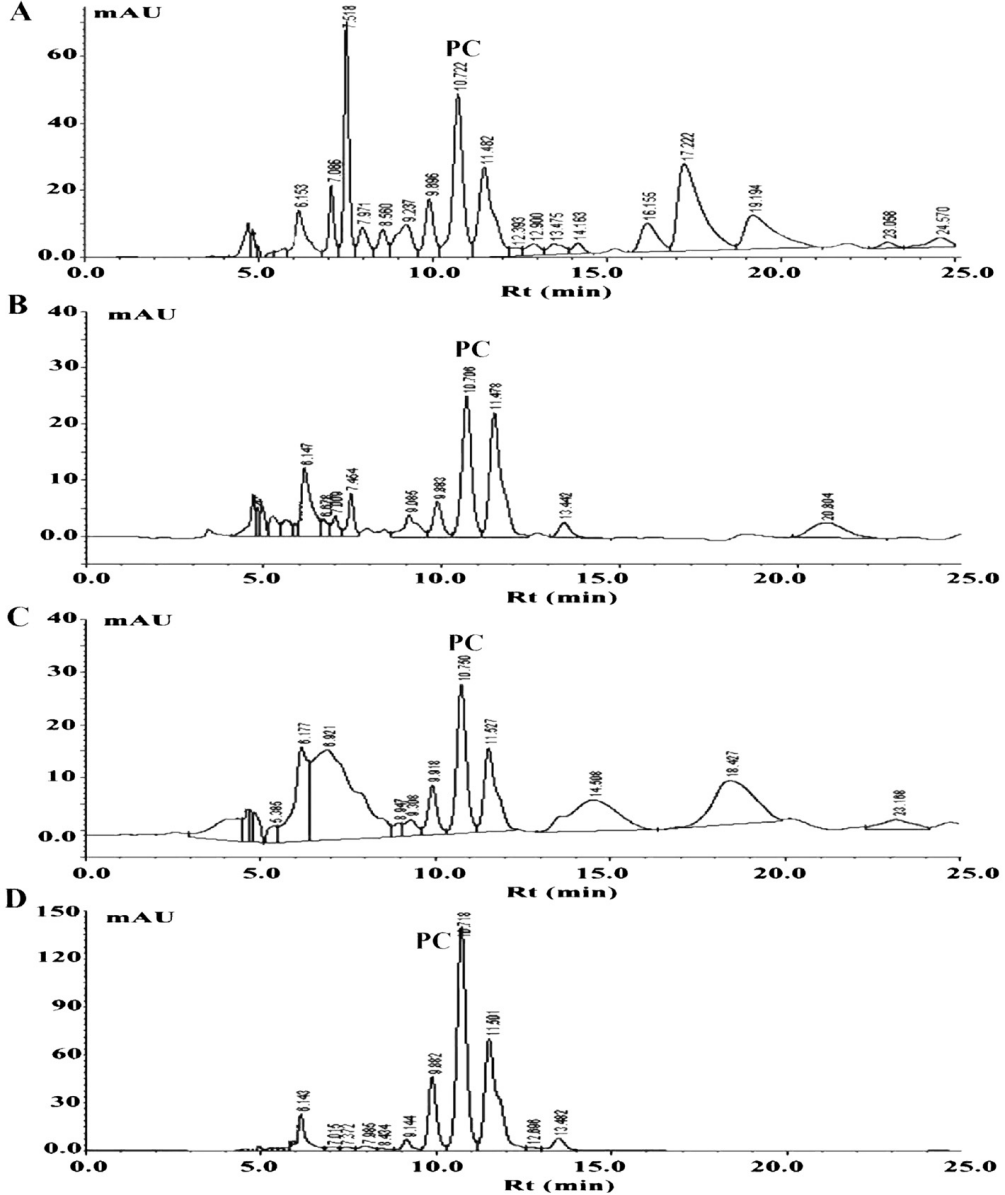
**HPLC chromatograms of different types of soybean phospholipids.** Crude soybean phospholipids **(A)**, soybean phospholipid extract prepared in ethanol (PH-Et) **(B)**, soybean phospholipid extract prepared in acetone (PH-Ac) **(C)**, and phospholipid fraction prepared by column chromatography (PH-Fr) **(D)**.

The crude soybean lecithin was found to contain 62 ± 0.2% acetone insoluble phosphatides, and HPLC analysis indicates phosphatidylcholine (PC) is the main phospholipid ingredient in the phospholipids prepared (Figure 2). The percentage of PC (based on peak area) in crude soybean phospholipids, soybean phospholipid extract prepared in ethanol (PH-Et), soybean phospholipid extract prepared in acetone (PH-Ac), and phospholipid fraction prepared by column chromatography (PH-Fr) was 13.4%, 21.6%, 10.6% and 42%, respectively.

**Figure 3:**
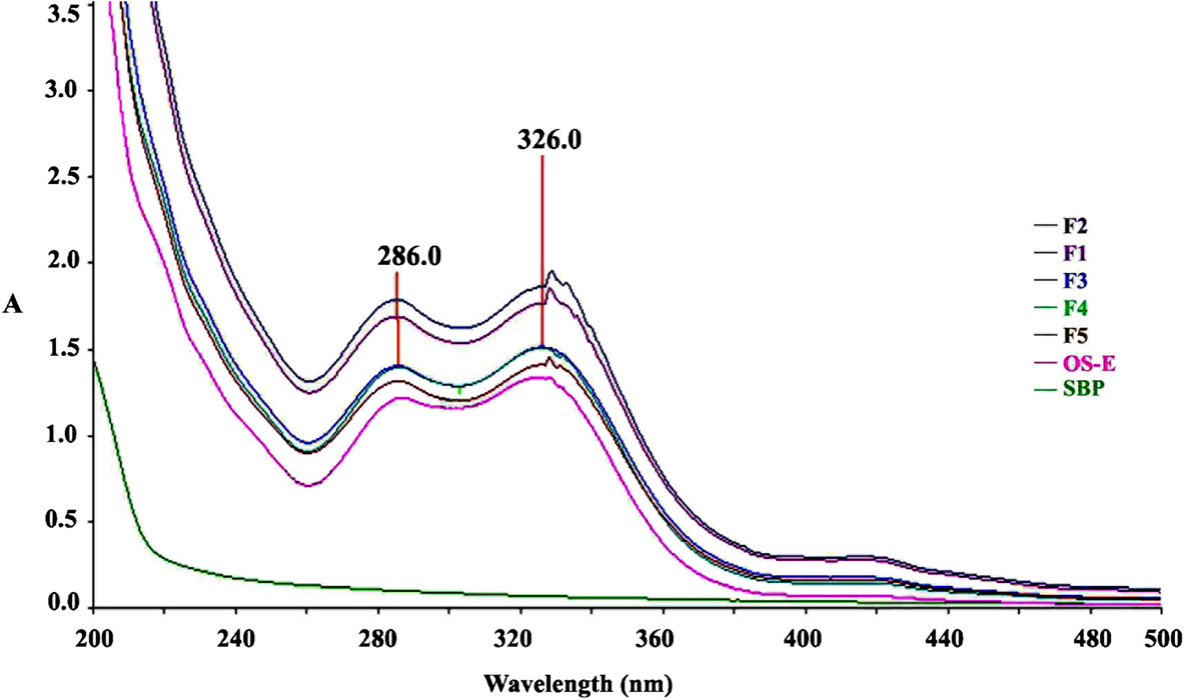
**UV–vis spectrophotometry of various formulations of OS ethanolic extract prepared in soybean phospholipids.** The OS-E extract showed maximum absorption at 286 nm and 326 nm in both formulated and non-formulated extract. OS-E refers to ethanolic extract, SBP refers to soybean phospholipid, and F1-F5 refers to different formulations prepared at the following phospholipids to extract (mg/mg) ratios: 150/50, 100/50, 50/50, 25/50 and 25/75, respectively. **(A)** refers to absorbance.

**Figure 4:**
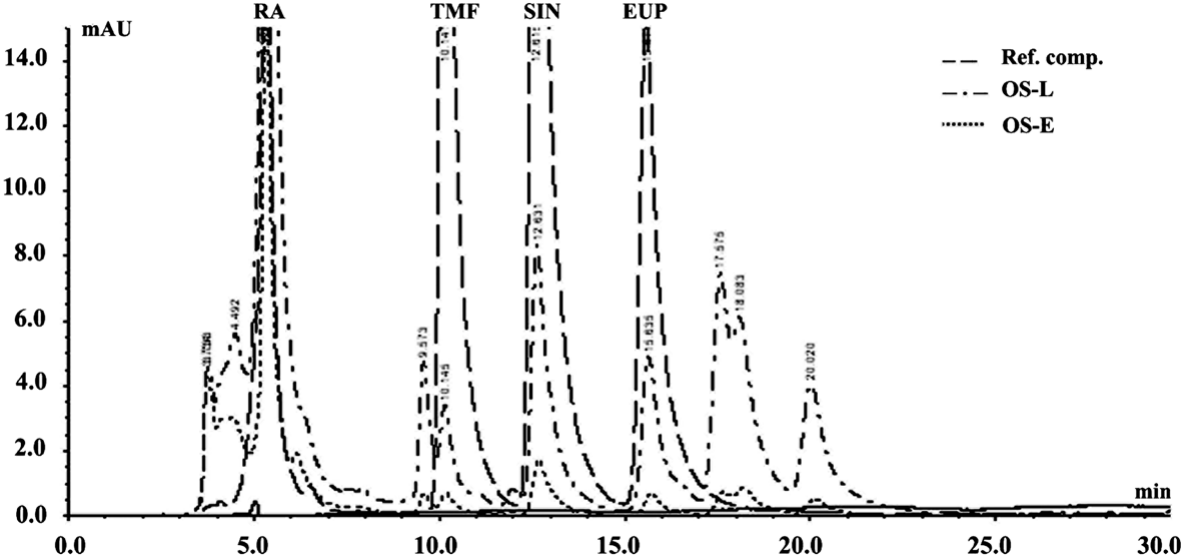
**HPLC chromatograms of OS ethanolic extract in formulated and non-formulated forms.** The chromatograms show presence of RA, TMF, EUP and SIN; the solubility of these compounds are improved significantly in the formulated OS extract compared to non-formulated extract. OS-E refers to *O. stamineus* extract, and OS-L refers to *O. stamineus* liposomes. RA, TMF, SIN and EUP refer to rosmarinic acid, 3′-hydroxy-5, 6, 7, 4′-tetramethoxyflavone, sinensetin and eupatorin, respectively.

Phytochemical analysis of OS extract revealed presence of high content of phenolics (26%) and glycosaponins (17%). UV–vis spectrum showed maximum absorption at 326 and 286 nm (Figure 3), and HPLC analysis (Figure 4) revealed presence of RA (2.2%), EUP (1.7%), SIN (0.23%) and TMF (0.1%).

### Optimization of preparation method

#### Selection of phospholipids to extract ratio

The optimum phospholipids to extract ratio was selected based on the improvement in aqueous solubility of OS extract. At first, the formulations were prepared using PH-Et phospholipid extract at various w/w ratios with OS extract. Solubility of nonformulated OS extract (OS-E) was determined by UV–vis spectrophotometry and was found to be 956 ± 34 μg/ml. The formulations were prepared by mixing ethanolic solution of the extract with chloroform solution of phospholipids followed by evaporation of solvents and hydration of the resulting film. Aqueous solubility results showed significant improvement in formulations prepared at phospholipid: extract ratio of 50:50, 150:50 and 100:50 (Table 1). The formulation prepared at 100:50 w/w ratio showed the highest improvement in solubility; therefore this formulation was selected for further optimization.

**Table 1:** Aqueous solubility of various OS liposomes

**No.**	**PH-Et (mg)**	**OS-E (mg)**	**Solubility (μg/ml)**	***P *** **value**
F1	150.0	50.0	1296.5 ± 18	0.000
F2	100.0	50.0	1401.7 ± 66	0.000
F3	50.0	50.0	1188.8 ± 169	0.001
F4	25.0	50.0	1018.8 ± 15	0.526
F5	25.0	75.0	1072.6 ± 6	0.084

Results are shown as AV ± SD, and the P value was calculated by One way ANOVA with Dunnett’s Post Hoc test versus extract’s solubility in water (n = 3).

#### Selection of solvent to dissolve the OS extract

At the beginning ethanol was selected to dissolve the extract since OS ethanolic extract is used in this study. However, solubility results of formulated extract were not very encouraging. Therefore, methanol was used to dissolve the extract. Aqueous solubility results showed substantial improvement of OS extract from 1402 ± 66 μg/ml (ethanol) to 3979 ± 139 μg/ml (methanol) (P = 0.000). A possible explanation of these results is that chloroform evaporates (boiling point 61.2°C) at higher rate than ethanol (bp, 78.4°C), leaving behind an ethanolic solution of the extract and causing precipitation of phospholipids, leading to phase separation and the formation of a non-homogeneous mixture of extract and phospholipids. On the other hand, chloroform and methanol evaporate at almost the same rate due to the narrow difference between their bp (bp of methanol = 65°C) leaving behind a homogeneous film of the extract and phospholipids. Consequently, this made the water dispersion of the mixture much easier and lead to substantial improvement in solubility.

#### Selection of phospholipids extract

Three formulation types were prepared in 3 phospholipid extracts including PH-Et, PH-Ac, and PH-Fr. The formulations were prepared at phospholipid: extract ratio of 100:50, and using methanol to dissolve the extract. Aqueous solubility was determined by UV–vis spectrophotometry, and the highest solubility (3862 ± 155 μg/ml) was obtained in the formulation prepared in PH-Et phospholipid extract. Formulation prepared in PH-Ac phospholipid extract showed a solubility of 2997 ± 367 μg/ml, and the lowest solubility was obtained when PH-Fr phospholipid fraction (highest in PC content) was used (2082 ± 62 μg/ml) (P = 0.000). Therefore, the final formulation was prepared in PH-Et phospholipid extract that was used in all subsequent analysis.

### HPLC analysis of OS liposomes

HPLC analysis of 4 marker compounds and 4 unknown compounds revealed significant improvement in their aqueous solubility compared to that obtained in non-formulated extract (P values = 0.000, Student’s t-test) (Table 2).

**Table 2:** HPLC analysis of 4 marker and 4 unknown compounds in OS extract (OS-E), and OS liposomes (OS-L)

**Compounds**	**RT (min)**	**OS-E (mAU)**	**OS-L (mAU)**
RA	5.3 ± 0.01	301.5 ± 1.3	1933.9 ± 7.7
TMF	10.2 ± 0.03	10.1 ± 1.7	67.5 ± 0.4
SIN	12.7 ± 0.02	49.5 ± 1.4	254.8 ± 1.0
EUP	15.7 ± 0.03	19.1 ± 1.6	148.8 ± 19.9
5	9.6 ± 0.01	10.4 ± 1.3	85.8 ± 0.8
6	17.6 ± 0.07	13.0 ± 1.3	194.5 ± 0.4
7	18.2 ± 0.08	14.8 ± 1.2	223.4 ± 16.0
8	20.1 ± 0.05	18.1 ± 0.1	145.5 ± 25.9

Results are shown as average peak area (mAU) ± SD (n = 3).

### Fourier transform infrared spectroscopy

**Figure 5:**
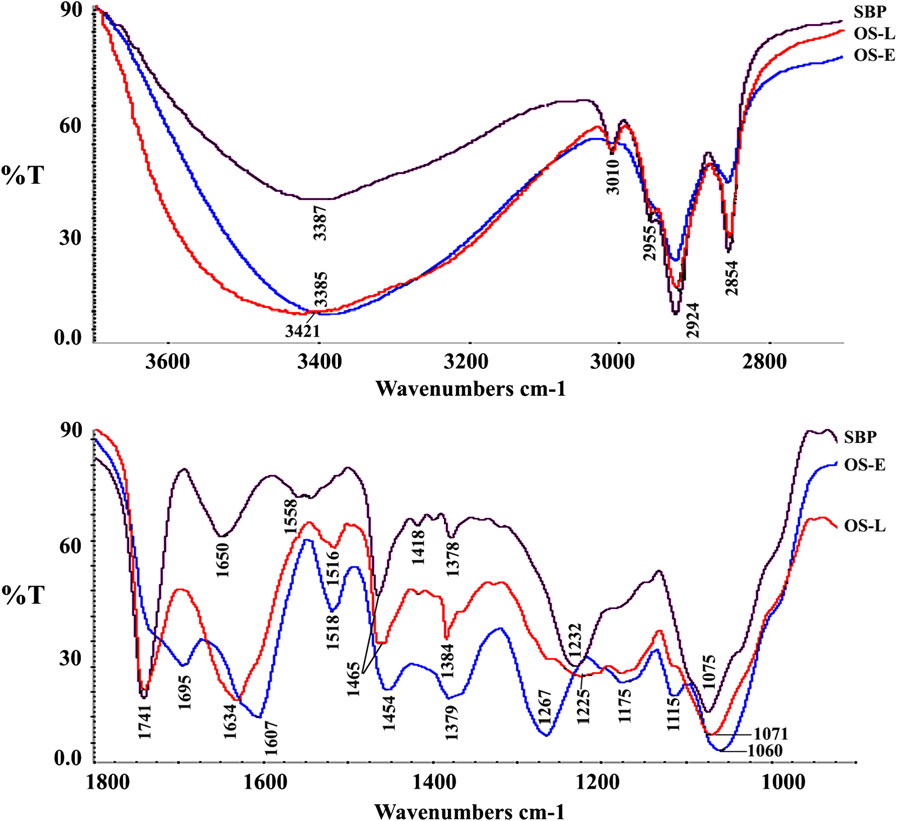
**FTIR spectra of *O. stamineus *ethanolic extract (OS-E), soybean phospholipids (SBP), and liposomes (OS-L).** Remarkable changes can be seen in the infrared absorption spectra due to incorporation of OS extract in phospholipids; the broad band corresponding to OH groups is shifted from 3387 cm^−1^ to 3421 cm^−1^, the C = O bands at 1695 cm^−1^ and 1607 cm^−1^ disappeared, the band corresponding to C = O stretching in phospholipids is shifted from 1650 cm^−1^ to 1634 cm^−1^, the band at 1267 cm^−1^ is shifted to 1225 cm^−1^, the band at 1232 cm^−1^ disappeared, and band at 1075 cm^−1^ is shifted to 1071 cm^−1^.

FTIR spectra of soybean phospholipid, OS extract, and OS liposomes were studied in order to get insights into occurrence of interaction between OS extract and phospholipids (Figure 5). In PH-Et phospholipids extract the broad band centered at 3387 cm^−1^ represents the OH stretching, the principal bands at 2854 cm^−1^ and 2924 cm^−1^ correspond to the symmetric and anti-symmetric stretching in the CH_2_ groups of alkyl chains, the strong band centered at 1741 cm^−1^ corresponds to the stretching vibrations of the ester carbonyl groups, the band centered at 1650 cm^−1^ is assigned to C = O stretching, and the scissoring vibrations of the CH_2_ groups are represented by the band at 1465 cm^−1^. The characteristic phosphate group vibrational band assigned to the PO_2_− anti-symmetric stretching mode is centered at 1232 cm^−1^ and the PO_2_− symmetric stretching mode PO_2_− at 1075 cm^−1^ , these results are similar to previously reported results [25]. In OS extract, the broad band centered at 3385 cm^−1^ corresponds to OH stretching, vibrations at 2924 cm^−1^ and 2854 cm^−1^ correspond to C-H stretching, the bands centered at 1695 cm^−1^ and 1607 cm^−1^ correspond to stretching vibrations of carbonyl groups, the bands at 1600–1420 cm^−1^ correspond to phenyl groups, the bands at 1267 cm^−1^ corresponds to ester carbonyl groups, and the band at 1060 cm^−1^ corresponds to primary OH groups. These results are consistent with previous reports [26].

Remarkable changes were observed in the infrared absorption spectra as a result of incorporation of OS extract in phospholipids; the broad band corresponding to OH groups is shifted from 3387 cm^−1^ to 3421 cm^−1^, the C = O bands at 1695 cm^−1^ and 1607 cm^−1^ disappeared, the band corresponding to C = O stretching in phospholipids is shifted from 1650 cm^−1^ to 1634 cm^−1^, the band at 1267 cm^−1^ is shifted to lower frequency (1225 cm^−1^) with much lower oscillation strength, the band at 1232 cm^−1^ disappeared, and band at 1075 cm^−1^ is shifted to 1071 cm^−1^. The most pronounced spectral changes can be observed in the spectral region corresponding to the polar heads of phospholipids. Such changes may represent hydrogen bonding between the polar heads of phospholipids with the hydroxyl groups in OS extract. Hydrogen bonding can also occur between the keto groups of flavonoids and oxygen groups of phospholipids. Hydrophobic interaction may also occur between the flavone’s methoxy groups and the phospholipid tails.

### Effect of pH on stability of liposomes

Stability of the OS liposomes at pH 1.6, 5.5 and 7.4 was studied in order to predict their stability in the gastrointestinal tract. Agglomeration and precipitation of OS liposomes occurred immediately after mixing with PBS at pH 1.6, but not at pH 5.5, or 7.4 or water. The percentage of the soluble fraction (stable), relative to that in water, was 66 ± 1.0% (pH 1.6), 94 ± 1.0% (pH 5.5) and 93 ± 3.0% (pH 7.4). These results indicate that OS liposomes are highly stable at pH 5.5 and 7.4, but relatively unstable at pH 1.6.

In order to verify whether the agglomeration observed at pH 1.6 is reversible, the precipitate obtained at this pH was re-dispersed in PBS at pH 1.6, 5.5 and 7.4. The precipitate was totally dissolved at pH 5.5 and 7.4 but not at pH 1.6. These findings indicate that the agglomeration observed at pH 1.6 is reversible at pH 5.5 and 7.4. Under GIT pH, the OS liposomes are expected to agglomerate under gastric conditions and re-dissolve under intestinal pH. It is noteworthy that soybean phospholipids and OS extract separately did not show any precipitate at pH 1.6.

### Entrapment efficiency

OS liposomes are unstable at pH 1.6, a property which was used to study the entrapment efficiency of OS liposomes. Entrapment efficiency was found to be 66.2 ± 0.9% (n = 3). This value is considered satisfactory when compared to published data on liposomal drug delivery systems of herbal extracts or compounds from herbal origin [15]. Entrapment efficiency of RA, TMF, SIN and EUP was 54.1 ± 0.2%, 28.2 ± 0.2%, 43 ± 0.2% and 38.3 ± 5.1% respectively. The average entrapment efficiency (EE) of the known fraction is almost 41%, as measured by HPLC. The known fraction of the extract (including TMF, RA, EUP and SIN) is only 4% of the total components, and the exact EE of the unknown fraction is unknown and cannot be measured accurately (theoretically, it can be higher than 66% for some compounds and lower for others). These two points may help to explain the difference in EE observed. Basically, the lipophilic compounds have poor solubility in water, and they have higher tendency to be entrapped in the lipid bilayers of liposomes. Whereas the hydrophilic drugs may be entrapped inside the aqueous cores of liposomes, or located in the external water phase [16]. It is noteworthy that the unentrapped extract can be removed by centrifugation after acid treatment of the liposomes, or by dialysis method without pretreatment, or can be kept in the formulation. In this study the free extract was kept in the formulation in order to avoid extract loss, and reduce the production expanses.

### The n-octanol: water partition

When a mixture of hydrophilic and hydrophobic compounds is partitioned between n-octanol and water, the hydrophilic compounds are distributed in water and the hydrophobic compounds are concentrated in n-octanol layer. Since OS liposomes have improved the extract’s solubility, it is expected that the OS extract will be more concentrated in the aqueous layer. Our results show that 40 ± 1.0% of the non-formulated extract is distributed in the aqueous layer which indicates relatively low hydrophilicity of the OS extract. On the other hand, this ratio is increased to 64 ± 1.0% in the OS liposomal formulation, which indicates increased extract’s hydrophilicity.

### Particle size analysis and transmission electron microscopy (TEM)

**Figure 6:**
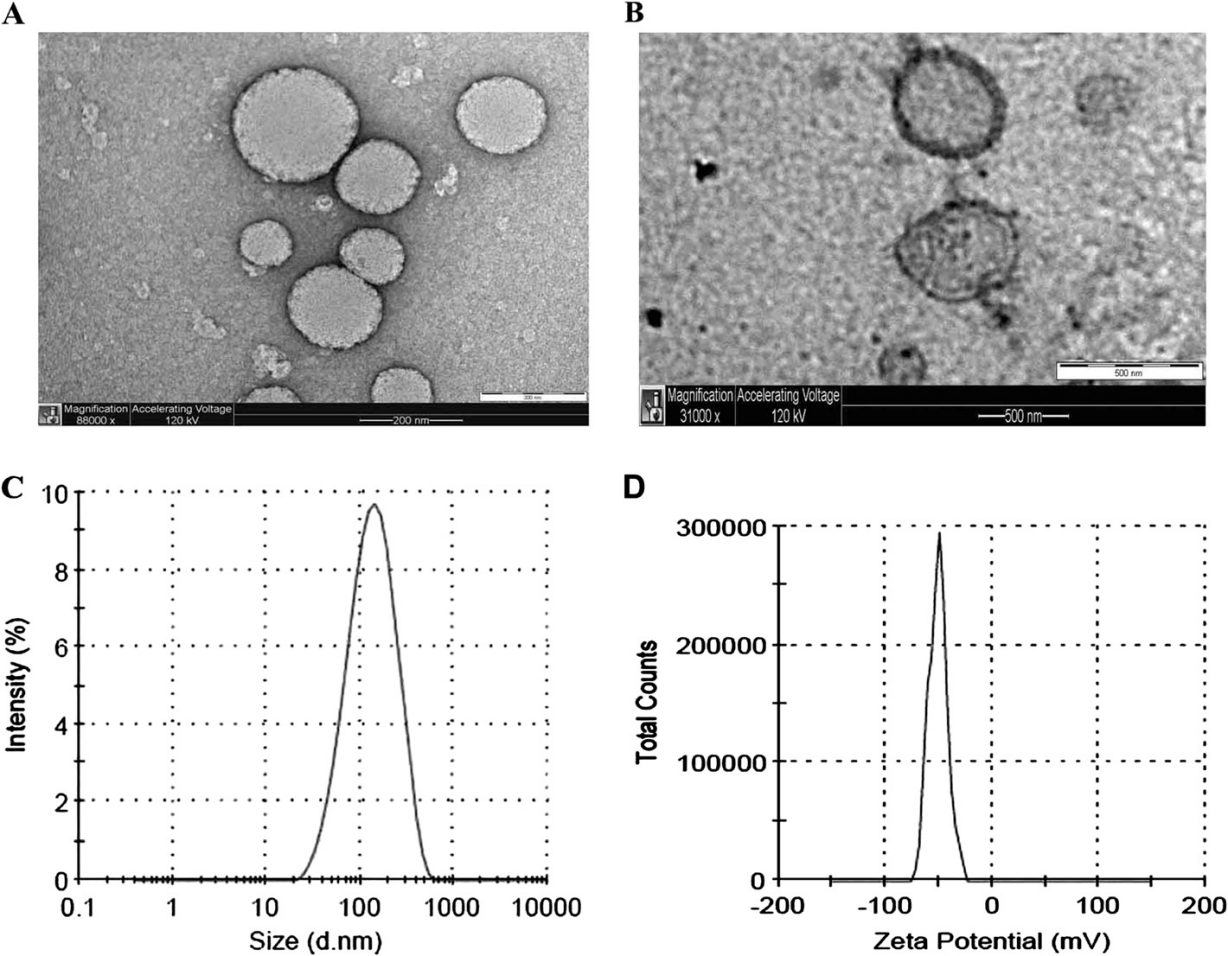
**Morphology and particle size analysis of the OS liposomes.** Transmission Electron Microscopy (TEM) photograph of OS liposomes **(A)** and their lipid bilayer **(B)**, particle size distribution **(C)**, and zeta potential distribution **(D)**. This figure confirms the presence of round liposomes with narrow size distribution (<200 nm) and a negative surface charge.

Particle size measurement by Photon Correlation Spectroscopy (PCS) indicates presence of structures with dynamic diameter of 153–177 nm with narrow size distribution. Zeta potential measurements showed presence of anionic liposomes with a surface charge from −40 to −49 mV (Table 3). The presence and morphology of liposomes were further confirmed by TEM which verified the presence of round liposomes of <200 nm. Furthermore, the lipid bilayer of the OS liposomes can be seen clearly at high magnification (Figure 6).

**Table 3:** Analysis of particle size and zeta potential by PCS

**Items**	**Particle diameter (nm)**	**Intensity (%)**	**Count rate (Kcps)**	**Polydispersity index (PdI)**	**Zeta potential (mV)**
SBP	177.2 ± 1.2	100.0 ± 0.1	543.8 ± 2.8	0.236 ± 0.004	−40.0 ± 2.2
OS-L	152.5 ± 1.1	99.0 ± 1.7	307.0 ± 17.3	0.233 ± 0.011	−49.8 ± 1.0

Results are displayed as AV ± SD (n = 3).

### Critical micellar concentration

**Figure 7:**
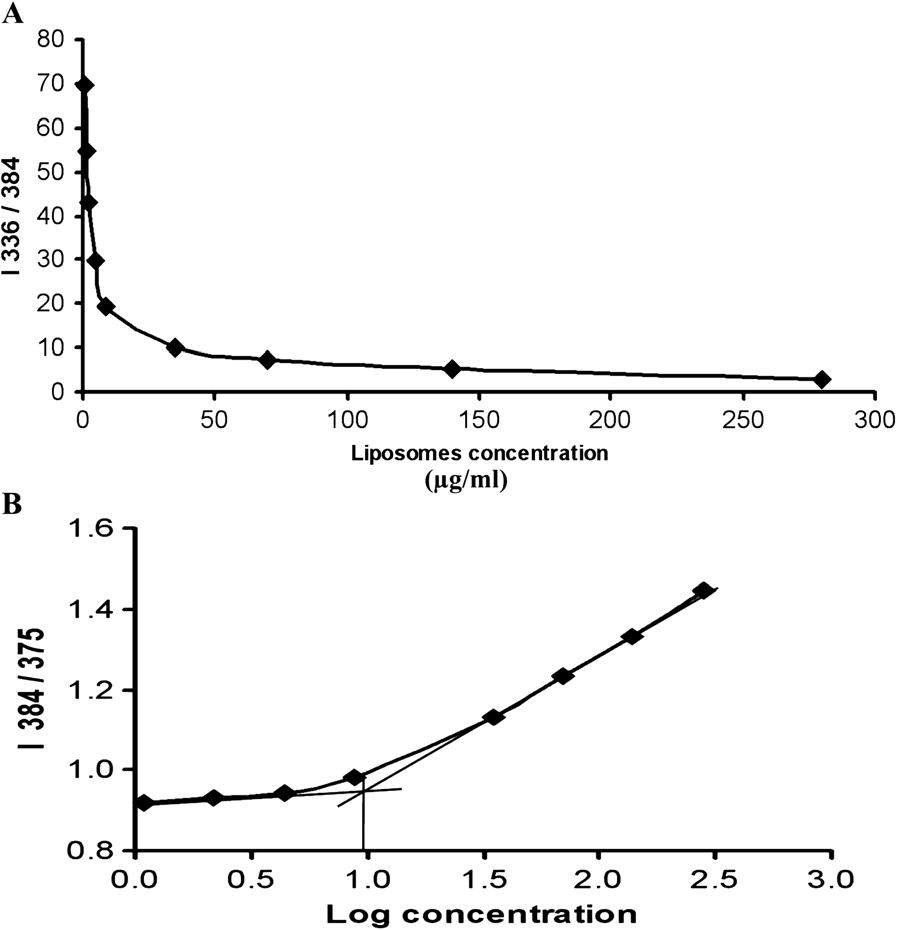
**Dose dependent quenching of pyrene fluorescence in OS liposomes.** Addition of pyrene to aqueous solution containing OS liposomes caused substantial quenching of pyrene’s fluorescence in a dose dependent manner **(A)**; increasing liposomes concentration caused a redshift in emission intensity and increased the I384/I375 intensity ratio **(B)**. The critical micellar concentration (CMC) is estimated from graph B and was found to be 9.2 ± 2.9 μg/ml.

The critical micellar concentration (CMC) was determined using the fluorescent probe pyrene. The results showed that addition of pyrene to aqueous solution containing OS liposomes caused substantial quenching of pyrene’s fluorescence in a dose dependent manner (Figure 7A). Increasing liposomes concentration also caused a redshift in emission intensity, and the intensity ratio I384/I375 of pyrene was increased accordingly. The CMC was estimated from the graph (Figure 7B) and was found to be 9.2 ± 2.9 μg/ml.

### In vitro release and antioxidant effect

**Figure 8:**
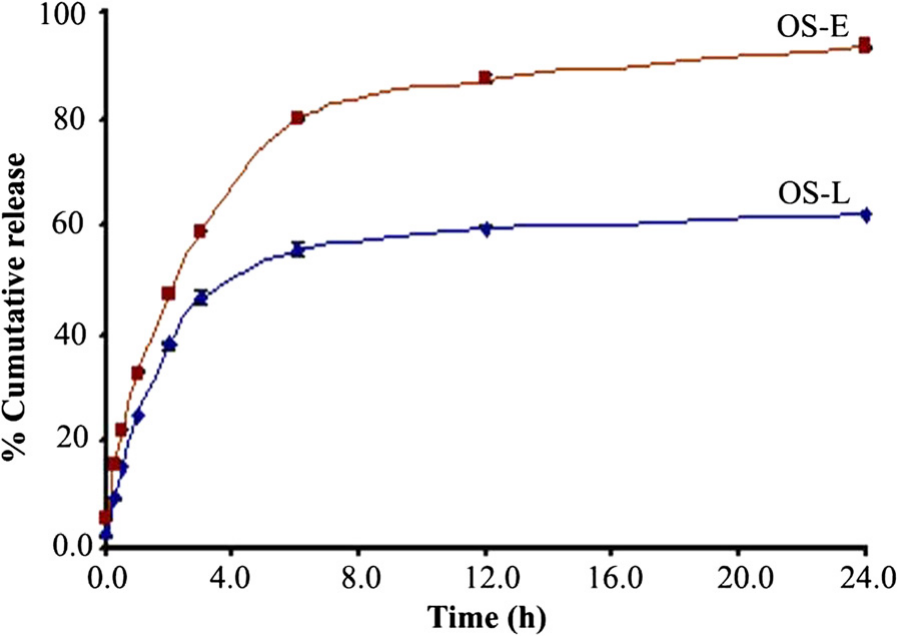
**Percentage cumulative release of OS liposomes (OS-L) and OS non-formulated extract (OS-E) at pH 6.8 and 37°C after 24 h.** Concentration of released extract was determined by UV–vis spectrophotometry. The percentage cumulative release in non-formulated extract was 94% and reduced to 62% in the formulated extract.

Despite of the immediate dissolution of OS liposomes in water, the percentage cumulative release was 62% after 24 h at 37°C and pH 6.8. On the other hand, the percentage cumulative release of non-formulated extract under the same conditions was 94% (Figure 8). This result gives more insights into stability of the liposomal structures under the test conditions, most likely due to the interaction between OS extract and the phospholipid carrier, and consequently leading slow release of the active principles. The released extract was then studied for DPPH scavenging effect. The results showed potent scavenging of DPPH in both OS liposomes and non-formulated extract; however with stronger effect obtained in OS liposomes (IC_50_ = 23.5 ± 1.1 μg/ml) than in non-formulated extract (IC_50_ = 32.4 ± 0.5 μg/ml), P = 0.000.

### Absorption through everted intestinal sacs

Absorption studies were carried out using everted rat small intestinal sacs under physiologic conditions (Tyrodes solution, pH 7.4 and 37°C and aeration). Intestinal sacs were recovered after 1 h incubation, and the contents were analyzed by HPLC. Significant improvement in absorption of 7 compounds was achieved, with a minor reduction in absorption of rosmarinic acid (RA). The largest improvement was obtained in eupatorin (EUP) which showed 9.8 ± 0.60 folds increment in the compound’s absorption (Table 4). These results indicate higher intestinal permeability of liposomal extract than non-formulated extract, which can be explained due to encapsulation in phospholipids, increased solubility, presence of nanoscale liposomes, increased negativity and colloidal stability [27]. Previous research on anionic liposomes has shown enhancement of colloidal stability and absorption [23], and even with higher cellular uptake rate than neutral and cationic liposomes [28,29].

**Table 4:** Absorption through everted intestinal sacs after 1 h incubation

**Compounds**	**RT (min)**	**OS-L/OS-E ratio**
Unknown 1	4.9 ± 0.04	3.0 ± 0.40
RA	5.3 ± 0.06	0.9 ± 0.01
TMF	10.0 ± 0.16	4.0 ± 0.70
SIN	12.3 ± 0.22	2.3 ± 0.20
EUP	15.2 ± 0.32	9.8 ± 0.60
Unknown 2	17.6 ± 0.37	3.6 ± 0.10
Unknown 3	19.4 ± 0.44	3.4 ± 0.20

Results are presented as the AV ± SD of absorption ratio of OS liposomes (OS-L) to non-formulated extract (OS-E) (n = 3–4).

## Conclusions

Collectively, the data presented in this study provide evidence about feasibility of using unpurified soybean phospholipids for preparation of nano liposomes of *O. stamineus* ethanolic extract. The OS liposomes demonstrated significant enhancement of intestinal absorption of the active principles, which can be attributed to the improvement in aqueous solubility and permeability, presence of nanoscale anionic liposomes, and colloidal stability of OS liposomes. The sustained release profile along with the improvement in free radical scavenging effect may provide a prolonged protection effect against oxidative stress related diseases. Therefore, this study may provide a basic formulation of *Orthosiphon stamineus* ethanolic extract for preparation of oral drug delivery system, and possibly topical delivery systems to the skin.

## Methods

### Preparation and standardization of *O. stamineus* ethanolic extract

OS dried leaves were obtained from specialized supplier of herbal products in Malaysia (Herbagus Trading, Kepala Batas, Pulau Pinang). OS ethanolic extract was prepared by the maceration method as the following; 100 g of OS powder was added to 1 L of 96% ethanol, mixed continuously on a magnetic stirrer for 48 h, filtered, concentrated at 60°C by rotavapor and further freeze-dried to 10.6 g of solid material. Total glycosaponins content of the extract was analyzed as previously described [30], and total phenolics content was estimated as previously described by Aisha et al., [31]. Concentration of 4 marker compounds including rosmarinic acid (RA), sinensetin (SIN), eupatorin (EUP), and 3′-hydroxy-5,6,7,4′-tetramethoxyflavone (TMF) was determined by reverse phase HPLC as previously described [14]; analysis was carried out using Agilent 1100 HPLC system, using Nucleosil C18 column (250 mm × 4.6 mm, 5 μm), column temperature was 25°C, injection volume was 20 μl, the mobile phase was isocratic which consisted of methanol (55%): tetrahydrofuran (5%): 0.1% H_3_PO_4_ (40%). Flow rate was 0.7 ml/min, separation time 25 min, and wavelength was 330 nm.

UV–vis spectrophotometry was carried out in the wavelength range 500–200 nm using Perkin Elmer UV spectrophotometer.

### Preparation of soybean phospholipids

The unpurified soybean phospholipids were prepared from food grade soybean lecithin as previously described with minor modifications [32]. In brief; crude lecithin (500 g) was refluxed in 96% ethanol (2.5 L) for 30 min and cooled to RT. Subsequently, the supernatant was collected by decantation and concentrated at 60°C using rotavapor. The residue was then washed 5× with acetone (1 L) to give 50 g of semi solid material; this phospholipid was called PH-Et. In another experiment, 500 g of the food grade lecithin was refluxed for 30 min in acetone (2.5 L), cooled down to RT, and the precipitate was saved and further washed with acetone (5×) to give 220 g and named PH-Ac. In addition, a fraction containing higher concentration of phosphatidylcholine was prepared as mentioned previously [32]; this phospholipid fraction was named PH-Fr.

The crude lecithin was characterized by measuring concentration of the acetone insoluble phosphatides and by RP-HPLC [20,32]. The soybean phospholipid fractions were analyzed by RP-HPLC as previously described with some modifications [32]; Agilent 1100 HPLC system was used, and separation was achieved on Agilent Eclipse C18 column (250 mm × 4.6 mm, 5 μm) at 35°C, the mobile phase was isocratic which consisted of isopropyl alcohol, methanol and water (70:22:8 v/v) at 0.5 ml/min. The samples were prepared in methanol at 1 mg/ml and filtered through 0.45-μm syringe filter, injection volume was 10 μl, and detection was done at 205 nm.

### Preparation of OS liposomes

Liposomes of OS extract (OS-L) were prepared by the film method as the following; soybean phospholipids was dissolved in chloroform and OS extract was dissolved in ethanol or methanol, the solutions were mixed, and the solvent was evaporated under vacuum using rotary evaporator at 45°C for 30 min, followed by drying in oven at 60°C for 1 h.

### Determination of aqueous solubility

Solubility was evaluated by UV–vis spectrophotometry; OS extract, and OS liposomes were dissolved at theoretical concentration of 4 mg/ml in deionized water, vortexed for 2 min, and sonicated for 10 min. Subsequently, the solutions were centrifuged at 8000 rpm and 25°C for 10 min. Concentration of OS extract in the supernatant was determined at 286 nm. A calibration curve was prepared at the same time in order to calculate concentration of soluble OS extract (y = 0.0135x – 0.0423, R2 = 0.9999). All experiments were carried out in triplicates. Concentration of 4 marker and 4 unknown compounds was also determined by HPLC as described previously.

### Effect of pH on liposomes stability

Effect of pH on liposome’s stability was studied as previously described with some modifications [33]. OS liposomes were dissolved in water at 4 mg/ml and further diluted at 1:4 ratio in phosphate buffered saline (PBS) at pH 1.6, 5.5, 7.4 and water, and incubated for overnight (16 h) at 37°C. Subsequently, the solutions were centrifuged at 8000 rpm and 25°C for 10 min, and concentration of OS extract in the supernatant was determined by UV spectrophotometry at 286 nm. The precipitate obtained at pH 1.6 was resuspended in PBS (pH 1.6, 5.5 and 7.4), vortexed for 5 min, centrifuged, and the supernatant was analyzed by UV spectrophotometry. The results are presented as percentage of soluble fraction relative to liposomes diluted in water.

### Determination of entrapment efficiency

Entrapment efficiency was determined by precipitation of OS liposomes at pH 1.6. It is noteworthy that this pH caused precipitation of OS liposomes, but not the free extract. The OS liposomes was dissolved in water at 4 mg/ml, sonicated for 10 min, and centrifuged at 8000 rpm for 10 min. The supernatant (1 ml) was added to the same volume of PBS at pH 1.6, mixed thoroughly by vortex, centrifuged at 8000 rpm for 10 min, the supernatant containing the free extract was decanted, the precipitate was washed with PBS (pH 1.6) and resuspended in 1 ml PBS (pH 7.4). After sonication for 10 min, the extract content was determined by UV spectrophotometry at 286 nm. Concentration of RA, TMF, SIN and EUP was measured by HPLC as described previously [14]. The entrapment efficiency was calculated as the following:

$$\begin{array}{l} \mathrm{Entrapment}\;\mathrm{efficiency}\;=\;(\mathrm{concentration}\;\mathrm{in}\;\mathrm{precipitate}\\ \;\;\;\;/\mathrm{theoretical}\;\mathrm{concentration})\\ \;\;\;\;\times \;100\;\left(n\;=\;3\right). \end{array}$$

### Partition between n-octanol and water

OS extract and OS liposomes were prepared in deionized water at 1 mg/ml and mixed with the same volume of n-octanol. The mixtures were then mixed by several manual inversions (30 times), allowed to settle for 4 h, and centrifuged at 5000 rpm for 5 min to allow separation of the 2 layers. Concentration of OS extract in both layers was determined by UV–vis spectrophotometry at 286 nm. The results are presented as the ratio of extract concentration in aqueous phase relative to that in n-octanol.

### Fourier transform infrared spectroscopy

FTIR analysis of OS extract, soybean phospholipids and OS liposomes was carried out using Spectrum 400 spectrometer (Perkin Elmer, USA). The IR spectra were recorded in the range of 4000 – 400 cm^−1^ (n = 3).

### Measurement of particle size and zeta potential

Particle size, polydispersity index (PDI) and zeta potential (ζ) were determined by Photon Correlation Spectroscopy (PCS) using a Zetasizer nano zs (Malvern Instruments Ltd, UK). The samples were dissolved in ultra pure water (18 MΩ) at 1 mg/ml and filtered through 0.45-μm syringe filters to remove any insoluble matter. Measurements were carried out in triplicates.

### Determination of critical micellar concentration

Critical micellar concentration (CMC) of OS liposomes was estimated using the fluorescent probe pyrene [33]. Pyrene was dissolved in dimethylsulfoxide (DMSO) at 10 mM and diluted in PBS (pH 6.8) to 10 μM. Concentration of liposomes was maintained in the range 1–280 μg/ml in PBS. Pyrene was added at a final concentration of 0.1 μM and incubated for 30 min in the dark at RT. Fluorescence intensity was measured at excitation of 336 nm and emission of 375 and 384 nm by LS 45 fluorescence spectrometer (Perkin Elmer, USA). The intensity ratio (I384/I375) was calculated and plotted versus log concentration. The resulting curve was used to calculate the CMC (n = 3).

### Transmission electron microscopy

Studies in transmission electron microscopy (TEM) were carried out in order to confirm presence of the liposomal structures. One drop of OS liposomes (1 mg/ml in water) was deposited on a 400 mesh copper grid coated with 5 nm layer of carbon, air-dried at RT for 3 min, and stained with 2% uranyl acetate for 1 min. The samples were dried and studied using CM12 TEM (Philips, Netherlands).

### In vitro release and DPPH scavenging effect

In vitro release study was performed by dialysis method using dialysis bags with molecular weight cut off value 8200 Dalton. The experiment was carried out using magnetic stirrer in PBS at pH 6.8, and 37°C with continuous stirring at 100 rpm. Briefly, 10 ml of OS liposomes or non-formulated OS extract (in water) containing 10 mg of extract was filled in dialysis bag. The bags were hermetically sealed and kept in the receiver compartment containing 200 ml of same medium. Samples (3 ml) were collected at 0, 0.25, 0.5, 1.0, 2.0, 3.0, 6.0, 12, and 24 h, and immediately replaced with 3 ml fresh medium. Concentration of extract was determined spectrophotometrically at 286 nm. The results are presented as average percentage of cumulative release ± SD (n = 3). DPPH scavenging effect of released extracts was then investigated as described previously [31]; DPPH (9 mg/100 ml) was added to the same volume of released extract, incubated at RT for 30 min, and absorbance was measured at 516 nm. Median inhibitory concentration (IC_50_) was then calculated from the dose response curves (n = 3).

### Absorption through the everted rat intestine

Absorption through everted rat intestinal sacs was carried out as described previously with some modifications [27]. Overnight fasted male Sprague–Dawley rats were anesthetized by diethyl ether and euthanized by cervical dislocation. The first two thirds of the small intestine were collected immediately after euthanasia, everted gently using a glass rod, washed thoroughly, and were kept in Tyrodes solution (NaCl, 8 g; KCl, 0.2 g; NaHCO_3_, 1 g; CaCl_2_, 0.2 g; MgCl_2_.6H_2_O, 0.1 g; NaH_2_PO_4_, 0.05 g; and glucose, 1 g dissolved in 1 L deionized water) at 37°C with aeration. The tissues were cut into 6-cm length, sealed at one end using surgical thread and filled with 1 ml aerated Tyrodes solution. The intestinal sacs were kept for 1 h in a medium containing OS liposomes or OS non-formulated extract (120 μg/ml) in Tyrodes solution at 37°C with continuous agitation at 100 rpm using magnetic stirrer and with continuous aeration with air. Subsequently, sacs were removed, washed thoroughly from exterior with water, the content was collected and centrifuged at 10000 rpm for 5 min, and the supernatant was analyzed by HPLC [14]. Peak area of the marker compounds was recorded, and the fold change in absorption was calculated by dividing peak area of compounds in OS liposomes by that of non-formulated extract (n = 3–4).

### Animals

Male Sprague–Dawley rats were obtained from USM animal breeding facility, and were allowed to acclimatize for one week before the experiment. Experiment was performed according to the guidelines of USM Animal Ethics Committee (Ref. No.: USM/Animal Ethics Approval/2012/ (78) (399)).

### Statistical analysis

Results are presented as average ± SD of triplicate experiments unless otherwise mentioned. Differences between groups were considered significant at P < 0.05 using Student’s t-test or One way ANOVA.

## Abbreviations

OS: *Orthosiphon stamineus*; FTIR: Fourier transform infrared spectroscopy; TEM: Transmission electron microscopy; DPPH: 2,2-Diphenyl-1-picrylhydrazyl; RA: Rosmarinic acid; SIN: Sinensetin; EUP: Eupatorin; TMF: 3′-hydroxy-5,6,7,4′-tetramethoxyflavone; PC: Phosphatidylcholine; PI: Phosphatidylinositol; PE: Phosphatidylethanolamine; PH-Et: Soybean phospholipid extract prepared in ethanol; PH-Ac: Soybean phospholipid extract prepared in acetone; PH-Fr: Phospholipid fraction prepared by column chromatography; UV–vis: Ultraviolet–visible; RP-HPLC: Reverse phase high performance liquid chromatography; PBS: Phosphate buffered saline; OS-E: *Orthosiphon stamineus* ethanolic extract; OS-L: *Orthosiphon stamineus* liposomes; PCS: Photon correlation spectroscopy; CMC: Critical micellar concentration; PDI: Polydispersity index; RT: Room temperature; SD: Standard deviation; IC50: Median inhibitory concentration.

## Competing interests

The authors declare that no competing interests exist.

## Authors’ contributions

AFAA developed the concept, designed and carried out experimental work, analyzed the results, and prepared the manuscript. ZI and AMSAM developed the concept and revised the manuscript in its final version. All authors read and approved the final manuscript.
